# The Treatment Based on Temporal Information Processing Reduces Speech Comprehension Deficits in Aphasic Subjects

**DOI:** 10.3389/fnagi.2017.00098

**Published:** 2017-04-11

**Authors:** Aneta Szymaszek, Tomasz Wolak, Elzbieta Szelag

**Affiliations:** ^1^Laboratory of Neuropsychology, Department of Neurophysiology, Nencki Institute of Experimental Biology of Polish Academy of SciencesWarsaw, Poland; ^2^Department of Psychology, SWPS University of Social Sciences and HumanitiesWarsaw, Poland; ^3^Bioimaging Research Center, World Hearing Center of Institute of Physiology and Pathology of HearingKajetany, Poland

**Keywords:** temporal information processing, aphasia, stroke, phoneme discrimination, speech comprehension, temporal treatment

## Abstract

Experimental studies have reported a close association between temporal information processing (TIP) and language comprehension. Brain-injured subjects with aphasia show disturbed TIP which was evidenced in elevated temporal order threshold (TOT) as compared to control subjects. The present study is aimed at improving auditory speech comprehension in aphasic subjects using a specific temporal treatment. Fourteen patients having deficits in both speech comprehension and TIP were tested. The Token Test, phoneme discrimination tests (PDT) and Voice-Onset-Time (VOT) Test were employed to assess speech comprehension. The TOT was measured using two 10 ms tones (400 Hz, 3000 Hz) presented binaurally. The patients participated in eight 45-min sessions of either the specific temporal treatment (*n* = 7) aimed at improving the perception of sequencing abilities, or in a non-temporal control treatment (*n* = 7) on volume discrimination. The temporal treatment yielded an improvement in TIP. Moreover, a transfer of improvement from the time domain to the language domain was observed. The control treatment did not improve either TIP or speech comprehension in any of the applied tests.

## Introduction

### Temporal Dynamics in Language Processing

The relationship between temporal information processing (TIP) and language has been discussed for many years. Both experimental data and everyday observations have emphasized the temporal dynamics of human speech. Numerous publications indicate the fact that our language communication is rooted in TIP (Pöppel, 1997). In the temporal structure of the speech signal, two major levels may be distinguished, i.e., the millisecond level reflected in temporal segmentation in short time intervals and multisecond level reflected in segmentation in longer intervals. These two temporal levels are controlled by a high- or low-frequency processing system, respectively (Szelag et al., 2004, 2011).

High-frequency processing within millisecond time range might be crucial to phonemic hearing defined as primary ability to analyze and synthesize speech sounds. Auditory comprehension relies heavily on such an ability. For example, information about place and manner of articulation in stop consonants is contained within time range of *ca.* 20–40 ms. Spectrographic analyses in many languages clearly indicate that rapid formant transitions in stop consonants (like “p”, “b”, “t”, “d” etc.) are limited to time ranges of up to *ca.* 40 ms. Structure of human articulatory apparatus prevents prolonged articulation of these consonants in fluent speech due to immediate switching to vowel sounds, which follow. These frames of temporal processing are of paramount importance to our study. However, low-frequency processing system concerns rather lexical selection and sentence production or reception.

The relationship between TIP in the millisecond range and language reception may be supported by neuroanatomical data. Some authors postulated an overlapping of brain structures involved in both TIP and language which are located mainly in the left temporal lobe comprising gyrus temporalis superior, gyrus temporalis medius and surrounding white matter (von Steinbüchel et al., 1999; Wittmann et al., 2004; Woo et al., 2009; Lewandowska et al., 2010; Oron et al., 2016).

### TIP Deficits in Language Disordered Population

Several studies showed that the patients with left-hemispheric brain lesions and aphasia, children with language learning impairment, as well as children or adults with dyslexia display deficient TIP, specifically, a disordered ability of sequential processing and temporal ordering. These deficits were reflected in elevated thresholds for identification of events presented in rapid succession and observed in significantly longer, as compared to the healthy controls, inter-stimulus interval (ISI) between two sounds, necessary to report correctly their temporal order (e.g., Swisher and Hirsh, 1972; Tallal and Piercy, 1973; Farmer and Klein, 1995; Tallal et al., 1998; von Steinbüchel et al., 1999; Wittmann et al., 2001, 2004; Fink et al., 2006; Szelag et al., 2014, 2015). In this study, we concentrated on such temporal deficits in post-stroke aphasic patients which were assessed with temporal order threshold (TOT), i.e., the shortest ISI at which subjects were able to report the order of two sounds correctly.

In these patients, TIP has been widely investigated in the previous studies. A brief review of reports in existing literature shows that aphasic patients displayed deficient TIP on different time levels (millisecond/multisecond), depending on aphasic symptoms they present. In patients with Wernicke’s aphasia disturbed time perception on millisecond level was reflected in declined temporal resolution in millisecond time range (von Steinbüchel et al., 1999; Wittmann et al., 2004; Sidiropoulos et al., 2010). Such ability is crucial for auditory comprehension. Temporal resolution in the millisecond time range in aphasic patients can be tested using the monaural stimulus presentation mode, where one click is presented to the left or right ear, followed by another click to the other ear with different ISIs separating these clicks. The other possibility is the binaural stimulus presentation mode where two tones of different frequencies are presented to both ears with various ISI in between. The former mode was more extensively explored in existing studies on aphasic population than the binaural one.

For example, Wittmann et al. (2004) using monaural mode confirmed temporal ordering deficits in patients with a damage to the Wernicke’s area, who required significantly longer ISI between two clicks presented successively to report their order correctly in comparison to the healthy subjects. In line with this evidence, our previous studies confirmed in Wernicke’s patients disturbed temporal order perception measured with such monaural presentation mode (Szelag et al., 2014). It is worth mentioning that historically, Efron (1963), as well as Swisher and Hirsh (1972) emphasized that deficits in TIP in aphasia were not related selectively to the auditory processing, but concerned also the other modalities. Furthermore, deficits on the multisecond time range were evidenced also in Broca’s aphasic patients who displayed relatively preserved auditory comprehension but nonfluent output of speech and agrammatism. For such disfluency pattern multisecond timing seems to be the crucial factor (Szelag and Pöppel, 2000; Wittmann et al., 2001; Kagerer et al., 2002).

Moreover, in our previous study it was proven that in aphasic subjects individual differences in severity of phoneme-identification and phoneme-discrimination impairments were correlated with elevated TOTs (Oron et al., 2015). In this report, we evidenced in 30 aphasic patients that the elevated TOTs for monaurally presented clicks correlated positively with correctness achieved in standard language tests involving: (1) higher linguistic functions (measured by Token Test); (2) phoneme discrimination (tested by phoneme discrimination test (PDT)); and (3) voicing contrast detection (measured by Voice-Onset-Time (VOT) test). The existence of such correlations indicated that poorer sequencing abilities were accompanied by poorer language performance.

### The Application of TIP in Neurorehabilitation

Based on the coexistence of TIP and language deficits, the Fast for Word (FFW) computer training program was developed to improve both the sequencing abilities and phoneme discrimination, resulting in ameliorated auditory comprehension. This program was first applied in children with language-learning impairment (Merzenich et al., 1996; Tallal et al., 1996). FFW was also applied in dyslexic children and adults for improving their language competency (Temple et al., 2003; Strehlow et al., 2006; Gaab et al., 2007; Lajiness-O’Neill et al., 2007). Nevertheless, other authors did not confirm the positive effects of FFW on some aspects of language competency, i.e., writing and reading (Agnew et al., 2004).

A pilot treatment based on the relationship between TIP and language was used for the first time in our laboratory in aphasic patients (Szelag et al., 2014). In this study, the patients were taught to recognize the order of paired clicks presented monaurally in rapid succession with decreasing ISI within consecutive pairs. The training combined eight 45-min long sessions (temporal training). In parallel, the control group was trained using the volume discrimination procedure without any temporal aspects. We reported that only temporal training yielded improved TIP. Thus, after such training patients recognized the order of clicks within a pair at significantly shorter ISIs than before the training. Moreover, a transfer of improvement was observed from the trained time domain to the language domain which remained untrained during the intervention period. Importantly, following such therapy patients committed significantly fewer errors in Token Test, PDT and VOT Test. In contrast, the non-temporal control training did not improve either the temporal order perception or auditory comprehension in any of the applied language skills.

Due to the limited literature evidence with regard to amelioration of auditory comprehension following temporal training in aphasic patients (Szelag et al., 2014, 2015), as well as importance of this issue for modern neurorehabilitation, we extended our early pilot prototyping therapy procedure and developed the complementary training protocol, using the modified stimulus presentation mode. There is an important difference between our pilot prototyping procedure (Szelag et al., 2014) and the procedure presented here. It concerns the specificity of the stimulus presentation mode during intervention. In the former case, monaurally presented clicks were delivered contrary to binaurally presented tones in the latter case. Based on our previous reports, there are some fundamental differences in performance of sequencing tasks using monaural clicks or binaural tones as the presented material (Szymaszek et al., 2009; Szelag et al., 2011). Whereas the monaural task (using click presentation) reflects rather pure temporal processing, the binaural one (tones presentation) involved beside such processing the frequency discrimination, labeling the perceived pitch in the inner speech, and abstract thinking. Therefore, it seems to be more sensitive to subjects’ age in comparison to the monaural one. Moreover, in the monaural mode considerable deterioration was observed beyond 60 years of age, whereas in the binaural one declined performance started much earlier, approximately at the age of 40 years (Szymaszek et al., 2009). These frequency discrimination problems did not reflect difficulties in peripheral hearing as the normal hearing was evidenced by screening audiometry applied during participant recruitment. In aphasic patients, in binaural task, the overlapping of temporal problems related to brain lesions and difficulties related to the specificity of the task was observed. In consequence, only patients relatively cognitively well-functioning (with more subtle post-stroke deficits) were able to perform the binaural task.

It seems that different neuronal processes are involved in binaural as compared to the monaural procedure. Considering the interpretation provided by some authors (Brechmann and Scheich, 2005; Rahne et al., 2008; Deike et al., 2010), we are of the opinion that two tones presented in rapid sequence could be integrated into one percept at short ISIs. Their temporal order therefore, can be reported on the basis of one frequency-modulated pattern (rising/falling) rather than on identification and sequencing of separate stimuli.

Some authors emphasized that the binaural presentation involved the integration of information from both hemispheres, as the left posterior part of the superior temporal gyrus is probably specialized for sequential tasks. Nevertheless, the participation of the right-hemispheric global strategy for categorization of pitch direction (global pattern recognition) was also confirmed (Johnsrude et al., 2000; Zatorre et al., 2002; Brechmann and Scheich, 2005). In our post-stroke subjects population only left hemispheric areas were disturbed, which in consequence may be the reason of such TIP deficits.

### Study Aims

Considering the above evidence, in the present study we investigated whether the deficits in temporal sequencing abilities in aphasic patients can be reduced by the specific temporal training using binaural tone presentation. Furthermore, we verified if such a training could be profitable for both TIP and language domains, as previously reported training using monaural presentation (Szelag et al., 2014).

## Materials and Methods

### Participants

Fourteen patients (nine males and five females) suffering from post-stroke, moderate to mild fluent aphasia after hemeorrhage or infarction, (lesion age ± SD = 18 ± 13 weeks) participated in the study. They aged from 40 to 77 years (± SD = 57.3 ± 11 years), were right-handed (Oldfield, 1971), Polish native speakers and had normal hearing level (ANSI, 2004) verified by a screening audiometry (audiometer AS 208), using frequencies of 250, 500, 750, 1000, 1500, 2000 and 3000 Hz, which covered the frequency spectrum of stimuli presented in the study. Apart from stroke they had neither neurological nor psychiatric disorders and reported no history of head injuries in the past. The other exclusion criteria were: multiple stroke, concomitant systemic diseases, poor general health, participation in other rehabilitation program during our study and older age to minimize age-related cognitive deficits which might have negative effects on the training results.

All recruited patients displayed auditory comprehension deficits. Three standard language tests were administered to assess comprehension abilities (see below). The language deficits were accompanied by disordered TIP, evidenced in auditory perception of temporal order.

The patients were classified randomly into two groups: experimental group (EXP) and control group (CON). These groups were matched for age, gender, type of stroke, lesion age, as well as for baseline characteristic measures tested in this study, i.e., the level of auditory comprehension, attentional resources (see below for description of particular procedures). All between-group differences for these variables were nonsignificant (Mann-Whitney *U* test). It was a randomized, single-blind study, thus, patients were not informed to which group they were assigned.

The EXP group (*n* = 7) was assigned to the experimental treatment program and the CON group (*n* = 7)^1^ to the control treatment program. The baseline characteristic of the study population is summarized in Tables 1, 2.

**Table 1:** **Baseline characteristics of the study population**.

	Experimental treatment group (EXP)	Control treatment group (CON)
Patient age (mean ± SD, years)	54.7 ± 13	59.3 ± 9
Lesion age (mean ± SD, months)	20 ± 15	16 ± 11
Type of stroke (infarction/hemorrhage ratio)	6/1	5/2

**Table 2:** **Detailed description of the patient sample assigned to group EXP and CON**.

No	Gender	Patient age (years)	Lesion age (weeks)	Type of stroke	Group
1	M	40.2	33	I	EXP
2	M	66.6	18	I	
3	M	57.8	12	I	
4	M	45.9	16	H
5	M	76.6	7	I	
6	M	45.5	6	I	
7	F	50.5	47	I	
8	M	63.6	5	I	CON
9	M	61.4	7	I	
10	M	48.6	36	I	
11	F	76	17	I	
12	F	57.4	8	I	
13	F	54.8	15	H	
14	F	52.5	24	H	

*Abbreviations: M, male; F, female; I, infarction; H, hemeorrhage stroke*.

**Figure 1:**
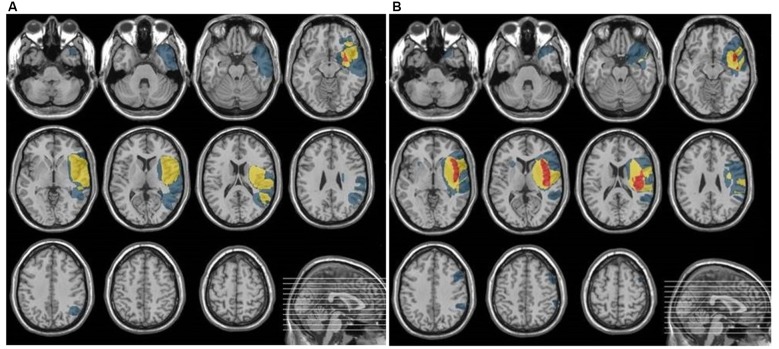
**The summarized lesioned areas in patients included into EXP group (A)** and CON group **(B)**.

The place of the lesion was verified by CT or MRI. Neuroanatomical analyses are summarized in Figure 1. which indicates overlapping of lesioned areas in both cortical and subcortical regions in EXP and CON groups. The lesioned areas in EXP group comprised mainly of: superior temporal gyrus, medial orbito-frontal cortex, basal ganglia: insula, putamen, whereas, in CON group: superior and middle temporal gyrus, Heschl’s gyrus, Rolandic operculum, medial orbito-frontal cortex and basal ganglia: insula, putamen.

As shown in Figure 1, the damaged structures in both groups comprised mainly temporal area of the left hemisphere which covered the classical regions involved in both receptive language function (auditory comprehension) and temporal processing (von Steinbüchel et al., 1999; Wittmann et al., 2004; Szelag et al., 2009). Considering the description of the patient sample presented above, the EXP and CON groups were fully matched.

The research was approved by the Bioethics Commission at the Institute of Psychiatry and Neurology in Warsaw (permission no. 5/2005 from February 2nd, 2005) as well as by the Bioethics Commission at the Warsaw Medical University (permission no. 5/2010 from January 26th, 2010). The study was conducted according to the principles expressed in the Helsinki Declaration; the written informed consent from each participant was obtained prior to testing.

### Experimental Procedures

The study combined both treatment programs and cognitive evaluation procedures. The treatment programs comprised of either the experimental treatment (EXP) or control treatment (CON). The cognitive evaluation procedures included language, attention and TIP.

#### Treatment Programs

##### Experimental treatment (EXP) program

The EXP program was focused on TIP. It used paired tones of different frequencies (low tone: 400 Hz and high tone: 3000 Hz) which were presented with different ISIs binaurally *via* headphones. After each paired-tone presentation, a patient reported the order of tones by pointing to a response card corresponding to *high-low* or *low-high* tone order. A starting ISI was the mean TOT value achieved in two sessions during *the baseline evaluation* (see below for detailed description) increased by 20 ms to ensure a comfortable treatment situation. In EXP an adaptive algorithm was applied to adjust the level of task difficulty for each patient individually (see below). EXP program consisted of 10-trial blocks. Within each block the paired tones were presented with the same ISIs, whereas between blocks the ISIs varied regarding an adaptive algorithm which based on correctness achieved in previous responses. There were the passed and the missed blocks. The *“passed block”* was successfully completed when at least 8 out of 10 trials within this block were answered correctly. Then, in the next block the ISI was shortened to make the task more demanding for the patient. The steps applied in ISI decreasing depended on the actual value of patients TOT. For TOT longer than 100 ms the step was 5 ms, whereas, for TOT from 50 ms to 100 ms, and for TOT below 50 ms it was 2 ms and 1 ms, respectively. On the other hand, in the *“missed block”* only seven or fewer correct responses within a block were given. In such a situation ISI in the next block was increased by a constant 1 ms step.

##### Control treatment (CON) program

In CON program two 1000 ms sounds separated by a constant, relatively long ISI of 3000 ms were presented (Szelag et al., 2014). The subject assessed the loudness of sounds and reported which sound was louder (the first or second) by pointing to a response card corresponding to the first and second sound. During CON treatment sounds of different frequency (400, 600, 800, or 1000 Hz) were presented in consecutive blocks, however, the frequency of sounds within a block (10 following trials) was always the same. The adaptivity in the control training was based on the loudness difference of paired tones. The loudness of the tones within a pair may differ within 0.025–0.00025 of the amplitude range with a constant step of 0.00025.

Both EXP and CON treatment programs were comparable as much as possible according to the task difficulty and the mental force. The only difference was in a lack of a millisecond TIP processing component which constituted a basic component of the EXP program.

The treatment protocol in each program consisted of eight training sessions performed three times a week. Each session lasted 45 min and comprised of 10-trial blocks. The same motivation system was used in EXP and CON treatment. Within the block each single trial was preceded by a visual warning signal. After each subject’s response the feedback on the correctness was provided. Each single correct response was rewarded by one point. Next, the points achieved were summarized and displayed to the patient at the end of each block. For each *“passed block”* the patient collected the puzzles to keep him highly motivated. The patients were allowed to make short breaks within the session, nevertheless the duration of pauses was excluded from the entire session time.

#### Cognitive Evaluation Procedures

##### Language

Auditory comprehension was assessed by the three following tests: (1) Token Test (Huber et al., 1983) to evaluate speech comprehension on a sentence level; (2) PDT (Nowak-Czerwińska, 1994); and (3) VOT Test (Szelag and Szymaszek, 2014) to assess phonemic hearing and comprehension deficits on single word level.

*Token Test* is a part of the Aachener Aphasie Battery and involves semantic, syntactic and/or post-interpretative processes. The test contains 20 plastic tokens varying in shape (squares and circles), size (small and large) and color (red, yellow, green, blue and white). The test consists of 50 commands classified into five sections of increasing difficulty. Examples of particular commands in consecutive sections are as follows: Section 1: Touch the red square; Section 2: Touch the big red circle; Section 3: Touch the green square and the red circle; Section 4: Touch the little red circle and the big yellow square; Section 5: Put the green square next to the red circle.

The *outcome measure* was the percent of errors committed in the entire test.

The percent of errors committed in the Token Test was used both as patients’ inclusion criterion and for evaluation (both for *the baseline* and *the post-treatment evaluation*).

*PDT* contains 64 paired-words, within these 75% included different and 25% the same words. The words within each pair differed in consonants, contrasted for the place of articulation, plosive, fricative, voicing or nasality.

The patient was asked to report whether words within the presented pair were the same or different. The responses were given by pointing to corresponding response cards. Subjects performed four series of eight paired words in each series. Alternative versions of the test were applied in the *baseline* and *post-treatment* evaluation.

The *outcome measure* was the percent of errors in the entire test.

*VOT Test* assesses the ability to differentiate between initial voiced and unvoiced stop-consonant in two Polish words /TOMEK/ and /DOMEK/ (*in English: Tom/house*) which differed in the initial voiced and unvoiced stop-constant (T or D). They were created by manipulation of the duration of an interval between the burst and the onset of laryngeal pulsing in the initial consonant in naturally uttered word /TOMEK/unvoiced. There were in total 21 pseudowords (synthesized using Adobe Audition software), presented randomly in six series. The VOT of the initial stop consonant varied from −100 ms to 90 ms in 10 ms steps, i.e., −100, −90, −80, −70, −60, −50, −40, −30, −20, −20, −10, 0, 5, 10, 20, 30, 40, 50, 60, 70, 80, 90 ms^2^. The patient differentiated categorically each presented word either as /TOMEK/or /DOMEK/ by pointing to the response card displaying either a picture of a boy or a house. Each subject performed 126 trials exposed in 6 series of 21 presentations of various VOT values.

As confirmed by our previous study on 67 healthy Polish volunteers (Szelag and Szymaszek, 2014), pseudo-words of the VOT from −100 ms to −70 ms are typically classified as a word /DOMEK/voiced and from 10 ms to 90 ms as an unvoiced word /TOMEK/. In between, i.e., for a VOT values from −60 ms to 5 ms there is a transition zone in which the probability of the discrimination of T or D is at a chance level.

The *outcome measure* was the percent of reported word /DOMEK/ for VOT of −100 ms to −70 ms and /TOMEK/ for 10–90 ms.

##### Attention

The evaluation procedure comprised two aspects of attention, i.e., vigilance defined as the ability to sustain or maintain attention over a longer period of time and alertness, defined the ability to raise and maintain a high level of attention in anticipation of a test stimulus (Test for Attentional Performance, Zimmermann and Fimm, 2007).

Vigilance: a low (440 Hz) and a high (1000 Hz) tone were presented sequentially in random order. The subject pressed a response pad when two identical tones (low or high) occurred in a row. The test lasted 10 min.

The outcome measure was the mean reaction time (RT) achieved in all test trials.

Alertness: simple RTs were measured in response to a visual stimulus (a white cross displayed in the screen center) which was preceded by an auditory warning signal. The subject pressed a response pad after each presentation of the cross. The test comprised 80 trials which were performed in four blocks: two blocks without any warning signal (A) and two blocks with an auditory warning signal (B). The blocks were administered according to the ABBA protocol.

The outcome measure was the mean RT achieved in all test trials.

##### TIP

The stimuli were pairs of 10-ms sinusoidal tones of 400 Hz and 3000 Hz (1-ms rise and fall time of each tone) presented in rapid succession with varied ISIs. Within each sequence the tones were adjusted to equal loudness on the basis of isophones^3^. The paired-tones were presented *binaurally*, i.e., each pair was presented to both ears. The stimuli were generated by a 16-bit Sound Blaster Extigy Card and delivered at a comfortable listening level through Sony headphones MDR-CD 480. The subject reported temporal order of tones presented within each pair by pointing to one of two response cards corresponding to *high-low* or *low-high* tone order. A warning signal of 400 ms-long preceded each stimulus pair and it was delivered 1500 ms before the first tone in each pair.

In each trial, ISIs were adjusted according to an adaptive maximum-likelihood-based algorithm and the ISIs varied from 1 ms to 600 ms. This procedure estimated individual TOTs as the minimum ISI between two tones at which their order was reported by a subject at 75% correctness (Levitt, 1971). TOT values were adjusted using “*Yet Another Adaptive Procedure*” (Mates et al., 2001) on the basis of the maximum likelihood parameter estimation. The evaluation procedure was continued until the individual TOT value was located with a probability of 95% inside a ±5-ms interval around the currently estimated threshold (Treutwein, 1995).

The proper experiment was preceded by a practice session in which the subject was presented with a high and low tone separately to familiarize with sounds. Next, the patient was presented with paired tones at relatively long constant ISI of 600 ms and asked to report their order. Such a practice session was completed when the pre-defined criterion of 11 correct responses in last 12 presentations was reached. Then the proper experiment started.

*The outcome measure* was the mean TOT from two sessions.

The TOT measurement was applied both in the evaluation phase and in subjects recruitment as one of the inclusion/exclusion criterion. The subject performed the task twice during two sessions with a break of a few days. In case of recruitment, the mean value of two sessions was compared to that of healthy volunteers matched for age. The cut off value for patient inclusion to the training was the TOT value of 1.5 SD above the mean value evidenced in healthy volunteers (Szymaszek et al., 2009; Szelag et al., 2011).

**Figure 2:**
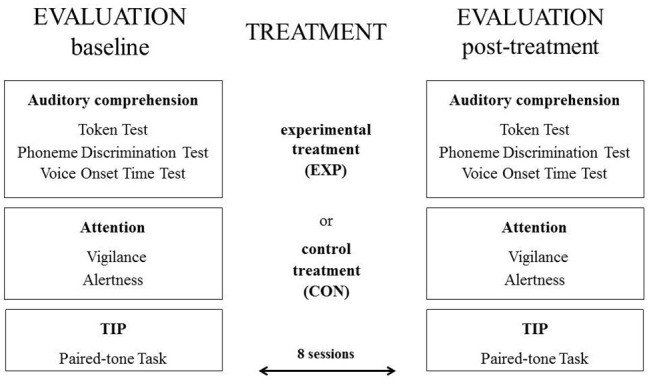
**The scheme of the experimental protocol**.

The cognitive assessment procedures were performed at the *baseline* and for the *post-treatment evaluation* (see Figure 2 below).

### Statistical Analyses

Using Wilcoxon Signed-Rank Test for two dependent samples, we compared the post-treatment vs. baseline performance in particular patients in two treatment groups. We tested the effect of EXP and CON treatment on language and attentional resources, as well as on TIP in sequencing abilities measured by the TOT.

## Results

### Language

#### Token Test

After EXP the difference in percentage of errors *post-treatment* ($\bar{x}$ = 33) vs. *a baseline* ($\bar{x}$ = 50) was significant (*n* = 7, *Z* = 2.37, *p* < 0.017). After CON the difference in percentage of errors *post-treatment* ($\bar{x}$ = 45) vs. *a baseline* ($\bar{x}$ = 50) was nonsignificant (*n* = 7, *Z* = 1.48, *p* < 0.14).

#### Phoneme Discrimination Test (PDT)

After EXP and CON the difference in percentage of errors *post-treatment* ($\bar{x}$ = 3 and $\bar{x}$ = 11, for EXP and CON, respectively) vs. *a baseline* ($\bar{x}$ = 7 and $\bar{x}$ = 14, for EXP and CON, respectively) was nonsignificant (*n* = 7, *Z* = 1.28, *p* < 0.21 and *n* = 7, *z* = 1.10, *p* < 0.28, for EXP and CON, respectively). Nevertheless, after EXP the percentage of errors *post-treatment* was lower than that at *a baseline*.

#### Voice-Onset-Time Test

After EXP in the typical unvoiced zone (VOT values of 5, 10 and 70 ms) the mean percent of correctly reported unvoiced pseudowords /TOMEK/ *post-treatment* was higher than *at a baseline*. It corresponded to the improved performance (VOT_5 ms_ = 91 vs. 81, *Z* = 1.83, *p* < 0.068; VOT_10 ms_ = 95 vs. 74, *Z* = 2.02, *p* < 0.044; VOT_70 ms_ = 96 vs. 86, *Z* = 1.83, *p* < 0.068). After CON the *post-treatment vs. baseline* difference for all VOT values was nonsignificant.

#### Attention

For both treatment programs there was no significant difference for the *post-treatment* and *a baseline* performance for both measured aspects of attention, i.e., alertness and vigilance.

For alertness the mean RT *post-treatment vs. a baseline* was $\bar{x}$ = 287 vs. $\bar{x}$ = 287 for EXP (*Z* = 0.11; *p* < 0.92) and $\bar{x}$ = 372 vs. $\bar{x}$ = 397 for CON (*Z* = 0.94; *p* < 0.35). For vigilance the mean RT *post-treatment vs. a baseline* was $\bar{x}$ = 613 vs. $\bar{x}$ = 645 for EXP (*Z* = 0.11, *p* < 0.92) and $\bar{x}$ = 584 vs. $\bar{x}$ = 536 after CON (*Z* = 0.54, *p* < 0.60).

#### TIP

After EXP the difference in TOT *post-treatment* ($\bar{x}$ = 120 ms) vs. *a baseline* ($\bar{x}$ = 257 ms) was significant (*n* = 7, *z* = 2.20, *p* < 0.028). After CON the difference the *post-treatment* TOT ($\bar{x}$ = 151 ms) vs. *a baseline* ($\bar{x}$ = 219 ms) was nonsignificant (*n* = 4, *z* = 1.46, *p* < 0.15).

**Figure 3:**
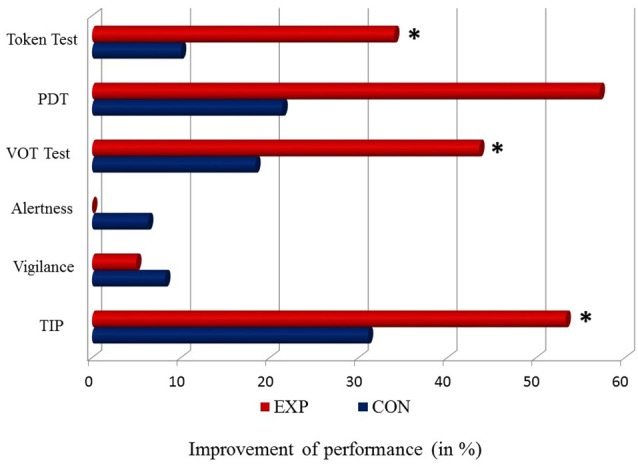
Percentage of differences in the level of a *baseline vs. post-treatment* performance (baseline—post-treatment/baseline * 100%) for particular tasks in EXP (red bar) and CON (blue bar). The 0 point reflects a stable performance (no difference between *a baseline vs. post-treatment* performance). Positive values (right side from the 0 point) correspond to improved performance. No worsened performance (left side from the 0 point) was observed. Significant differences (*p* < 0.05) are indicated by asterisks.

The results of EXP and CON treatment for all tests described above are presented in Figure 3.

## Discussion

### Summary of Results

The application of EXP training in aphasic patients significantly ameliorated TIP, which was reflected in lower TOT values (better performance) as well as language comprehension evidenced in Token Test, VOT test and PDT. In contrast, the CON training caused no significant improvement in any of the tests used. Moreover, attentional functions (alertness and vigilance) remained stable following both EXP and CON programs. Thus, the obtained language benefits resulted rather from the improvement of linguistic processes but not from changes in attentional resources after the treatment.

### Influence of EXP and CON Treatment Programs on TIP and Language Abilities

The study reported the effects of two different treatment programs (EXP and CON) on TIP and language competency in post-stroke patients suffering from aphasia following damage to the posterior parts of the left hemisphere. Although CON treatment did not cause any significant improvement, EXP treatment provided significant benefits for both language and TIP skills.

The application of EXP treatment resulted in significantly lowered thresholds for the order detection in *post-training vs. baseline evaluation*, corresponding to improved sequencing ability. In contrast, *post-training vs. baseline* difference turned out nonsignificant following CON treatment. Despite the similar mental load of these two treatment types, the huge advance of the EXP program was evidenced. It was rooted in improved, rapid sequencing processing which proved to be crucial for our cognition (Szelag et al., 2015).

Our previous preliminary study (Szelag et al., 2014) also revealed that temporal training was beneficial for aphasic subjects despite the use of different stimulus presentation mode from that applied in the present study. Both studies (Szelag et al., 2014 and presented here) indicated that the beneficial effects seem related to temporal aspects of auditory processing independently of the stimulus types applied for the improvement of TIP, unless each of them contains rapid auditory processing stimulation. Nevertheless, it is worth mentioning that in case of treatment procedure based on tone differentiation severely disturbed patients were not able to differentiate between high and low tones and in a consequence could not perform the training.

With respect to receptive language functions, EXP program seems to be very profitable, as compared to the CON treatment. There is a strong literature support for a close relationship between the millisecond timing and language (Oron et al., 2015, see also “Introduction” Section). The disturbed sequencing abilities on millisecond time range may be associated with problems in speech perception, which is temporally segmented in this range, i.e., phoneme discrimination. The coexistence of timing deficits and speech comprehension problems was proven previously in different language pathologies e.g., dyslexia, specific language-learning impairments and aphasia. On the basis of this evidence, some authors postulated the existence of central mechanism which controls TIP in the range of some tens of milliseconds (responsible for correct identification of temporal order of two events) as well as phoneme discrimination. It uses spectral cues which comprise rapid formant transitions in millisecond time window, similar to that critical for TIP on the millisecond range. As already proven (Szelag et al., 2014), the intervention based on temporal order detection in the millisecond time domain, lowered patients’ TOT, resulting in speech abilities improvement.

In the present study, temporal treatment program caused improvement in different language functions, i.e., in global speech comprehension (Token Test), as well as in the differentiation of voiced/unvoiced contrast (VOT test). The remarkable lowering of errors was also observed in PDT, however, this improvement did not reach the statistical significance. Since VOT test is focused on phoneme discrimination, the important result of the present study revealed that temporal treatment lowered the number of errors in Token Test, which measured the comprehension of spoken commands of increasing length and complexity. The functions measured in Token Test required not only well preserved linguistic processing (phonemic, semantic and syntactic) but also efficient verbal working memory which was not assessed in the present study. Nevertheless, different bibliographical data emphasize that working memory may be improved after temporal treatments (Szelag et al., 2015). It seems important because post-stroke cognitive disabilities may not be related selectively to language disturbances but also to the other cognitive functions, i.e., working memory, executive functions, etc.

The follow-up diagnosis performed 6 months after the treatment completion indicated relative stability of the observed improvements.

### Temporal Trainings as Promising Perspective for Neurorehabilitation

Our important finding is the indication that temporal treatment is beneficial for amelioration of receptive language functions. In the modern neurorehabilitation this is a promising idea of a method supporting the classic speech therapy. Neuropsychologists emphasized that one of the advances of temporal treatment is that during the therapy aphasic patients do not face directly their language problems (the treatment used nonverbal stimulation) which may motivate them to work and simultaneously foster improvement of untrained language competency.

Numerous studies indicate that TIP constitutes a basic process, which is at the roots of our mental activity in its broad aspect and covers not only language, but also other cognitive functions. von Steinbüchel and Pöppel (1993) introduced modern functional taxonomy, which distinguishes content-related functions (“what”) from logistic functions (“how”). Content-related functions represent mental content of human subjective experience, including but not limited to perception, memory, language, while logistic functions fund the base of mental activity, thus being superior to content-related functions. These scholars postulate that TIP might be a logistic function, which determines other functions of human cognitive process. Such a theory was further developed in our previous work (Szelag et al., 2015).

Some authors indicated the internal timing mechanism controlling perception of temporal order in millisecond time range. This mechanism may be related to neural gamma band oscillations with a periodicity of ca. 40 Hz which means that one oscillation period has *ca.* 25 ms duration (Oron et al., 2015). Pöppel (1997) asserted that the correct perception of tow sound order is possible when they occur at least in two successive oscillatory periods. We can assume that such mentioned hypothetical mechanism might be disturbed in brain lesioned patients, e.g., after a stroke resulting in longer periodicity of gamma oscillations and less efficient TIP.

### Final Remarks

To sum up, our recent results allow some generalizations to be drawn. Only temporal treatment improved both TIP and receptive language functions which was evidenced by the transfer of improvement from the trained time domain to untrained language domain. Such transfer was not observed after the control treatment. These results show the possible influence of TIP treatment on language deficits reduction in aphasic population.

## Author Contributions

AS: subject recruitment, acquisition, analysis and interpretation of data, manuscript writing. TW: analysis and graphical presentation of neuroanatomical data. ES: acquisition, analysis and interpretation of data, manuscript writing, responsibility for the final version of manuscript.

## Funding

The study was supported by Ministry of Science and Higher Education grant No: PBZ-MIN/001/PO5/06 realized in cooperation with researchers from the Human Science Centre, Ludwig-Maximillian University, Bad Tölz (Germany).

## Conflict of Interest Statement

The authors declare that the research was conducted in the absence of any commercial or financial relationships that could be construed as a potential conflict of interest. The reviewer TSG declared a shared affiliation, though no other collaboration, with several of the authors, AS and ES, to the handling Editor, who ensured that the process nevertheless met the standards of a fair and objective review.

## Footnotes

^1^A part of results in the CON group reported here was used as controls in our previously published study (Szelag et al., 2014).

^2^The negative VOT values reflect a situation when a burst is preceded by laryngeal pulsing (in Slavonic languages perceived usually as a voiced contrast), whereas, the positive VOT values reflect the opposite situation, i.e., pulsing is preceded by a burst (unvoiced contrast).

^3^http://www2.sfu.ca/sonic-studio/handbook/Phon.html
